# Structure and evolution of protein interaction networks: a statistical model for link dynamics and gene duplications

**DOI:** 10.1186/1471-2148-4-51

**Published:** 2004-11-27

**Authors:** Johannes Berg, Michael Lässig, Andreas Wagner

**Affiliations:** 1 Institut für Theoretische Physik, Universität zu Köln, Zülpicherstr. 77, 50937 Köln, Germany; 2 University of New Mexico, Department of Biology, 167A Castetter Hall, Albuquerque, NM 817131-1091, USA

## Abstract

**Background:**

The structure of molecular networks derives from dynamical processes on evolutionary time scales. For protein interaction networks, global statistical features of their structure can now be inferred consistently from several large-throughput datasets. Understanding the underlying evolutionary dynamics is crucial for discerning random parts of the network from biologically important properties shaped by natural selection.

**Results:**

We present a detailed statistical analysis of the protein interactions in *Saccharomyces cerevisiae *based on several large-throughput datasets. Protein pairs resulting from gene duplications are used as tracers into the evolutionary past of the network. From this analysis, we infer rate estimates for two key evolutionary processes shaping the network: (i) gene duplications and (ii) gain and loss of interactions through mutations in existing proteins, which are referred to as link dynamics. Importantly, the link dynamics is asymmetric, i.e., the evolutionary steps are mutations in just one of the binding parters. The link turnover is shown to be much faster than gene duplications. Both processes are assembled into an empirically grounded, quantitative model for the evolution of protein interaction networks.

**Conclusions:**

According to this model, the link dynamics is the dominant evolutionary force shaping the statistical structure of the network, while the slower gene duplication dynamics mainly affects its size. Specifically, the model predicts (i) a broad distribution of the connectivities (i.e., the number of binding partners of a protein) and (ii) correlations between the connectivities of interacting proteins, a specific consequence of the asymmetry of the link dynamics. Both features have been observed in the protein interaction network of *S. cerevisiae*.

## Background

Molecular interaction networks are ubiquitous in biological systems. Examples include transcription control [1], signal transduction, and metabolic pathways [2]. These networks have become a focus of recent research, because of their important roles in metabolism, gene expression, and information processing. Data on such networks are rapidly accumulating, massively aided by high-throughput experiments. Some of these networks are suffciently complex that their characterization requires statistical analysis, an area of considerable recent interest [3-5]. One key issue in this area is the distinction between structures reflecting biological function and those arising by chance. To address this issue requires an understanding of the biological processes that shape the network on evolutionary time scales. More precisely, one has to identify the statistical observables containing specific information about the evolutionary dynamics that shape a network.

In this paper we focus on protein interaction networks, whose nodes correspond to proteins, and whose links correspond to physical interactions between two proteins. Several complementary experimental techniques have been used to analyze pairwise protein and domain interactions, as well as protein complexes, in genome-scale assays [6-13]. Common to these approaches is a high rate of individual false negative and false positive interactions [14,15]. Different protein interaction data sets thus differ in many ways, but they also reveal similar *aggregate *(or global) network features, such as the fraction of nodes with a given connectivity. This implies that only large-scale statistical features of protein interaction networks can currently be reliably identified by high-throughput approaches. We here present an empirically grounded model that explains empirically observed statistical features of such networks.

**Figure 1:**
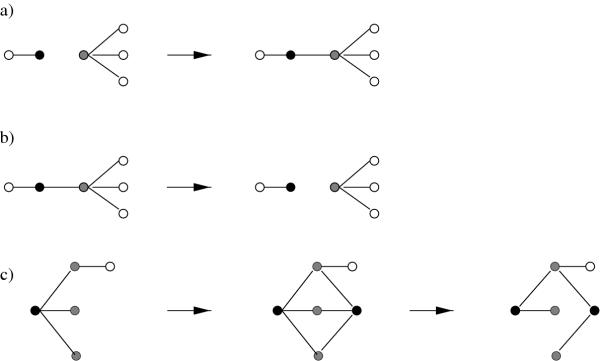
**The elementary processes of protein network evolution. **The progression of time is symbolized by arrows. **(a) Link attachment **and **(b) link detachment **occur through nucleotide substitutions in the gene encoding an existing protein. These processes affect the connectivities of the protein whose coding sequence undergoes mutation (shown in black) and of one of its binding partners (shown in gray). Empirical data shows that attachment occurs preferentially towards partners of high connectivity, cf. fig. 3. **(c) Gene duplication **usually produces a pair of nodes (shown in black) with initially identical binding partners (shown in gray). Empirical data suggests duplications occur at a much lower rate than link dynamics and that redundant links are lost subsequently (often in an asymmetric fashion), which affects the connectivities of the duplicate pair and of all its binding partners [22,25,38].

The currently best characterized protein interaction network is that of the baker's yeast *Saccharomyces cerevisiae*. On evolutionary time scales, this network changes through two processes, illustrated by figure 1. These are (i) modifications of interactions between existing proteins and (ii) the introduction of new nodes and links through *gene duplications*. Duplications of a single gene result in a pair of nodes with initially identical binding partners. Segmental and global duplications of the genome lead to the simultaneous duplication of many genes. On the other hand, processes affecting the interactions between *existing proteins *are referred to as *link dynamics*. Link dynamics results primarily from point mutations leading to modifications of the interface between interacting proteins [16]. Both kinds of processes, link dynamics and gene duplications, can be inferred from a statistical analysis of the network data, and their rates can be estimated consistently with independent information.

Of course, proteome function *in vivo *is influenced by further factors, notably gene regulation, which determines the concentrations of the proteins interacting in a living cell. The very definition of a bound state depends on the concentrations of the binding partners: A pair of proteins which binds at high concentrations may no longer form a bound state at lower concentrations. Here we concentrate on protein interactions at constant concentrations as they can be inferred from high-throughput datasets.

Previous work by others [17-19] shows how structural features of the network can in principle be explained through mathematical models of network evolution based on gene duplications alone. (For similar duplication-based models of regulatory and metabolic networks, see [20,21].) However, the overall rate of link dynamics has been estimated from empirical data in [22] and is at least an order of magnitude higher than the growth rate of the network due to gene duplications. It must therefore be included in any consistent evolutionary model.

In this paper, we present a model of network evolution that is based on observed rates of link and duplication dynamics. At these rates, the model predicts that important structural features of the network are shaped solely by the link dynamics. Hence, the evolutionary scenario of our model is quite different from the duplication-based models [17-19]. The statistical network structure predicted by the model is in accordance with empirical observations, see the discussion below.

This paper has two parts. In the first part, we estimate the rates of link attachment and detachment from empirical data. Specifically, we do not just estimate average rates of link dynamics for the whole network, because this has been done previously [22], but we show how the dependence of link attachment and detachment rates depends on the connectivities of both nodes (proteins) involved. (The connectivity of a protein is defined as the number of its interaction partners). We find evidence that the basic rate of link attachment is *asymmetric*. That is, this rate increases with the connectivity of only one of two the nodes involved. This reflects an asymmetry in the underlying biological process: a new protein-protein interaction is typically formed through a mutation in only one of two proteins.

In the second part of the paper, we assemble the estimated rates of link dynamics into a model of network evolution. Unlike for most other cases studied so far [3,4], the dynamics of these networks cannot be written as a closed equation dependent on the *connectivity distribution*, i.e. the fraction of nodes with a given number of neighbors. Instead, the analysis of networks under asymmetric link dynamics involves the *link connectivity distribution*, defined as the fraction of links connecting a pair of nodes with given connectivities.

The model has only one free parameter, the average connectivity of nodes in the network. Its stationary solution correctly predicts statistical properties observed in the data. Central properties of this solution are *connectivity correlations *between neighboring vertices, in accordance with recent observations in high-throughput protein interaction data [23]. These correlations are a consequence of the asymmetric link attachment process.

## Results and discussion

### Estimates of evolutionary rates

Two kinds of processes contribute to the evolutionary dynamics of protein interaction networks. The first consists of *point mutations *in a gene affecting the interactions of the encoded protein. As a result, the corresponding node may gain new links or loses some of the existing links to other nodes, as illustrated in fig. 1(a) and 1(b), respectively. We refer to these *attachment *and *detachment *processes, which leave the number of nodes fixed, as *link dynamics*. The second kind of process consists of *gene duplications *followed by either silencing of one of the duplicated genes or by functional divergence of the duplicates [24-26]. In terms of the protein interaction network, a gene duplication corresponds to the addition of a node with links identical to the original node, followed by the divergence of some of the now redundant links between the two duplicate nodes; see fig. 1(c).

Individual yeast genes have been estimated to undergo duplication at a rate of the order of 10^-2 ^per gene and per million years [27]. Some 90% of *single *gene duplicates become silenced shortly after the duplication, leading to an effective rate *g *of duplications one order of magnitude lower, i.e., ~ 10^-3 ^per million years [22,25,27,28]. Only a fraction of the yeast proteome is part of the protein interaction network, and gene duplicates involving proteins that are not part of the network do not contribute to its growth. Hence, *g *~ 10^-3 ^per million years should be considered an upper bound for the growth rate of the protein interaction network by gene duplications. A crude lower bound for the link attachment rate is *a *~ 10^-1 ^new interaction partners per node and million years. For instance, [22] estimated the rate at which new interactions were formed as no less than 294.5 new interactions per million years and approximately 1000 proteins. (These estimates are based on the formation of physical interactions between products of duplicate genes, and the approximately known age of the duplicates [22]. Importantly, most of these new interactions form between old duplicates, duplicates that are no longer under the relaxed selection pressure that is characteristic of young duplicates.) The above estimate gives a number of new interaction partners per protein per million years of *a *= 2 × 294.5/1000 = 0.589, five times greater than the lower bound of 0.1. To maintain an average network connectivity at the empirically observed value *κ *≈ 2.5 interaction partners per protein [25,29], the link detachment rate d has to be close to *a*, thus *d *~ *a *~ 10^-1 ^per million years. This rate of link attachment and detachment is much larger than the duplication rate of *g *~ 10^-3 ^per protein and million years. Hence, the link dynamics is decoupled from the much slower duplication dynamics. On intermediate evolutionary time-scales, the network reaches a stationary state of the link dynamics, while its number of nodes does not change significantly. This stationary state determines the structural statistics of the network, in particular the distribution of connectivities. On long time-scales, however, the network may grow through duplications. We emphasize that all these evolutionary rates are order-of-magnitude estimates, and that such estimates are suffcient for our model and the conclusions we derive from it.

**Figure 2:**
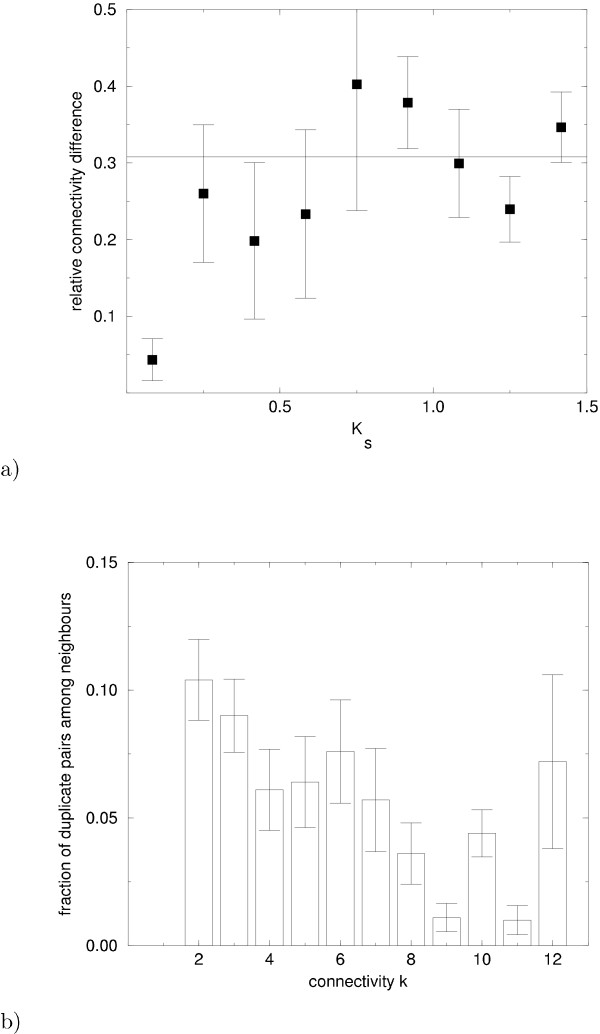
**(a) Duplicate protein pairs lose their connectivity correlations over time. **The average relative connectivity difference |*k *- *k*'|/(*k *+ *k*') of duplicate pairs with connectivities *k*, *k*' > 0 is plotted against the time since duplication, parameterized by the synonymous (silent) nucleotide divergence *K*_*s*_. The horizontal line indicates the value expected for two randomly chosen nodes. The average number of duplicate pairs per bin was 16 (from low values of *K*_*s *_to high ones the number of duplicate pairs per bin were 12, 5, 3, 6, 6, 8, 13, 27, 44 respectively). **(b) Duplications do not strongly influence network structure. **The histogram shows the fraction of duplicate pairs among the *k*(*k *- 1)/2 neighbor pairs of a node of connectivity *k *plotted versus *k*. A high number of duplicate pairs would be expected if duplications were a significant mechanism of link gain, see text. The mean and the standard error of this fraction were determined using proteins which are products of duplicate genes with sequence similarity *K*_*a *_< 1. The number of vertices used per column ranges from 374 for *k *= 2 to 8 for *k *= 12.

One basic but important empirical observation about link dynamics is the fast loss of connectivity correlations of proteins encoded by duplicate genes. Fig. 2(a) shows this loss, as estimated from empirical data. Specifically, the figure shows the average relative connectivity difference |*k *- *k*'|/(*k *+ *k*') of duplicate protein pairs as a function of the time since duplication, parameterized by the fraction *K*_*s *_of synonymous (silent) nucleotide substitutions per silent site. (As an order of magnitude estimate, a value of *K*_*s *_= 0.1 corresponds to a duplication age of 10 million years [25,27].) In the shortest time interval after duplication, the connectivities are still measurably similar. Soon thereafter, however, the relative connectivity difference becomes statistically indistinguishable from that of a randomly chosen pair of nodes, indicated by the horizontal line in fig. 2(a). Hence, diversification after duplication is a rapid process, with a time constant of the order of several 10 million years, consistent with the fast rate of link dynamics discussed above. An additional empirical observation underscores the minor importance of gene duplication in shaping the observed network structure. In models of network evolution based on gene duplication [17-19], a protein acquires new links through duplications of its neighbors (see, for example, the grey nodes in fig. 1(c)), at a rate proportional to its connectivity. This mechanism would generate an abundance of high-connectivity nodes. In addition, it would also generate a high fraction of pairs of neighbors that are products of a gene duplication. This is also true for intermediate models, incorporating both gene duplications and link dynamics, provided the duplication rate is comparable to the rate of link dynamics, or exceeds it. However this prediction of models based on gene duplications is not supported by the data. Fig. 2(b) shows the fraction of duplicate protein pairs among the *k*(*k *- 1)/2 neighbor pairs of a node of connectivity *k*. This fraction is small and it does not increase significantly with *k*. The data in this figure are also consistent with the earlier observation that the majority of duplicate pairs have few or no interaction partners in common [25].

We note that in our discussion of node dynamics we have not separately considered the effects of ancient genome duplications [39,40]. The conclusion that gene duplications do not shape the statistical features of the protein interaction network applies both to single gene duplications and to genome duplications. Indeed, the analysis of duplicates presented in figure 2 includes both pairs of genes resulting from single duplications and those stemming from genome duplications. Furthermore, the evolutionary dynamics of individual duplicated genes is similar for the products of single genome and whole genome duplications. For example, individual gene duplicates are lost with approximately the same probability in single duplications and in whole genome duplications. For this reason we do not, at this stage, include genome duplications separately in our model.

### Dependency of attachment rates on connectivities

The total rates *a *and *d *at which links are attached and detached in a protein interaction network allow no inference of how these processes shape the statistical properties of the network. To make such an inference, one must also know how the link dynamics depends on the connectivities of the nodes involved. The simplest possibility is that link attachment rates *a *and detachment rates *d *are functions of a node's connectivity *k*. The rates *a*_*k *_and *d*_*k *_at which links are attached or detached from a node of connectivity *k *have been estimated previously using interactions between products of duplicate genes [22]. They increase approximately linearly with *k*.

In representing attachment and detachment rates (*a*, *d*) as functions of connectivity *k *(*a*_*k*_, *d*_*k*_), one assumes implicitly that that the mechanism of link attachment and detachment is identical (symmetric) for the two nodes involved in a changed link. Previous analyses of protein network evolution [22] as well as models of network evolution [30] were based on such a symmetric process. However, the biological mechanism underlying link dynamics is intrinsically asymmetric. When a new link is formed, typically only one node undergoes a mutation, whereas the other node remains unchanged. This asymmetry means that the rate of link dynamics will generically depend in one way on the connectivity of the node undergoing mutation, and in another way on that of the unchanged node. As a result the rates *a*_*k *_and *d*_*k *_of link attachment and detachment are insuffcient to describe the dynamics of the network, since these rates will be different depending on whether the node is undergoing a mutation or not. This observation motivates the following estimate of the dependency of the link dynamics rate on node connectivities.

We define *a*_*k*,*k*' _as the probability per unit time that a given non-interacting *pair of proteins *with respective connectivities *k *and *k*' will acquire a link, multiplied by the number of proteins *N*. Analogously, we define the detachment rate *d*_*k*,*k*' _as the probability per unit time that a given interacting pair of proteins with respective connectivities *k *and *k*' will lose their link. The scaling convention of both rates is chosen such that the average connectivity of the network remains constant as the number of nodes *N *increases: the number of nodes pairs (where a link may be added) is proportional to *N*^2^, whereas the total number of links (which may be deleted) is proportional to *N*. We refer to the special case where the rates factorize, i.e. *a*_*k*,*k*' _~ *a*_*k*_*a*_*k*'_, as symmetric attachment (and analogously for the detachment rates *d*_*k*,*k*'_). The specific form of these rates assumes that link dynamics is a *local process*, so the probability for the formation or destruction of a link depends on the connectivities of only the two proteins involved in this process.

We now explain how one can estimate the dependency of *a*_*k*,*k*' _on its arguments, *k *and *k*'. As described earlier [22], one can use the observed number of physical interactions among duplicate gene products (cross-interactions) to estimate attachment rates. Briefly, such cross-interactions may arise in two ways. First, a protein that forms homodimers (a self-interacting protein) may undergo duplication, leading to two identical self-interacting proteins which also interact with each other. If both self-interactions are subsequently lost *independently*, yet the interaction between the nodes is retained, a cross-interaction is formed. This scenario does probably not account for the majority of cross-interactions, because it is inconsistent with data suggesting that self-interactions do not get lost overly frequently after duplication [22]. The second avenue of forming interactions between duplicate gene products involves a non-homodimerizing protein that undergoes duplication. Subsequently, an interaction between the duplicate proteins may form. If this mechanism is dominant, as we argue, one may use the number of cross-interactions to obtain order-of-magnitude estimates of the attachment rate [22]. From the number of interactions that each of the two involved proteins has with other proteins, one can estimate how the attachment rate depends on *k *and *k*'. The main caveat of this approach is that the connectivity of the duplicates may have changed since the time the link between them was formed.

**Figure 3:**
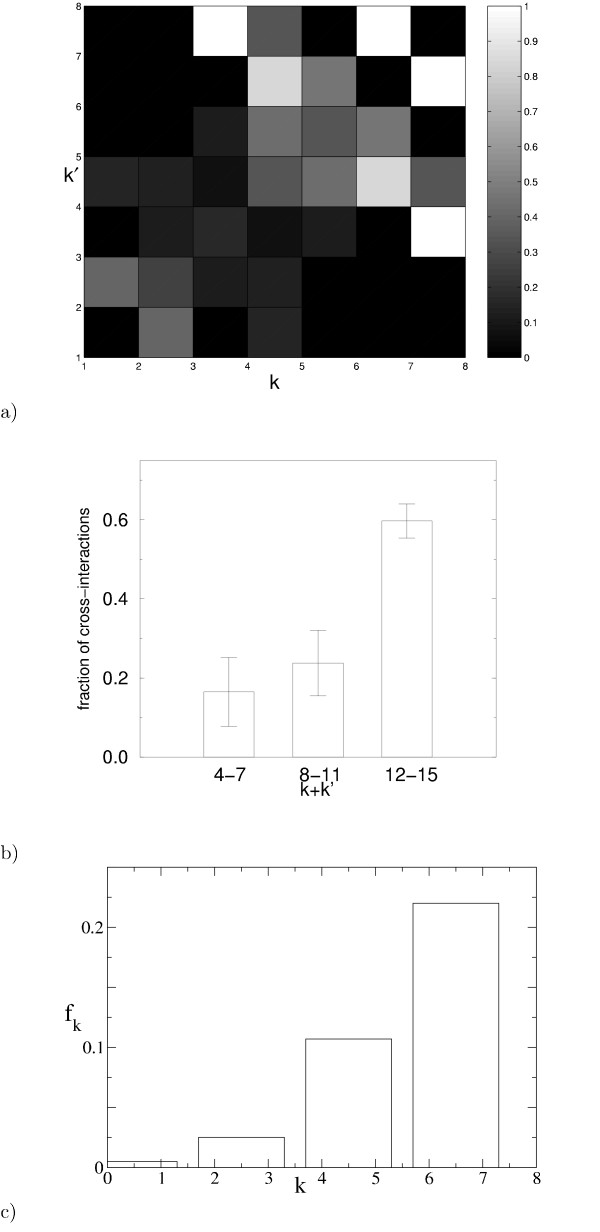
**Link attachment occurs preferentially towards proteins of high connectivity. (a) **The color-coded plot shows the fraction of duplicate pairs with connectivities (*k*, *k*') that have gained a mutual interaction (cross-interaction) since duplication, as a function of *k *and *k*'. Points where all duplicate pairs have cross-interactions are shown in white, points where none carry a cross-interactions are shown black. Points (particularly at high connectivities) where no data is available are also shown in black. The number of duplicate pairs with given connectivities ranges from 2 to 39. Points in the *k*, *k*'-plane where only a single pair of duplicates exists are excluded. **(b) **For this histogram the data from a) are binned for low, medium, and high *k *+ *k*' and the average for each bin is shown against *k *+ *k*'. The number of *k*, *k*' values contributing to each bin are 10, 14, and 11, from left to right. Error bars give the standard error. **(c) **Assuming the functional form *f*_*k *_+ *f*_*k*' _for the probability of a cross-interaction between nodes with connectivities *k *and *k*' (asymmetric attachment), the most likely values of *f*_*k *_may be deduced from the data (see text). The maximum-likelihood result shows an approximately linear increase of *f*_*k *_with *k*. The alternative scenario, symmetric attachment, yields a smaller maximum likelihood. Only duplicate pairs with *K*_*a *_≤ 0.4 were used in this analysis in order to avoid overcounting of cross-interactions of duplicates of even older duplicates.

The result of this procedure is shown in fig. 3. The sample size of 38 cross-interactions is extremely limited, but suffcient to demonstrate an increase of the attachment rate along the diagonal *k *= *k*', and no systematic change along other directions. A different representation of the same data in fig. 3b) also shows an increase of the attachment rate consistent with *k *+ *k*'.

An attachment process where one node with connectivity *k *is chosen with a probability , and a second one is chosen with probability , gives an attachment rate . The attachment rate *a*_*kk*' _~ *k *+ *k*' which we observe empirically is thus explained by an *asymmetric attachment process *where one node is chosen uniformly at random ( = constant), and the other node is chosen with a probability proportional to its connectivity ( ~ *k*). Note that the rate *a*_*k*,*k*' _itself is symmetric under interchange of the labels *k *and *k*', since either of the two nodes may take on the role of being preferentially chosen. However, the rate *a*_*k*,*k*' _does not factorize, exactly as required for an asymmetric attachment process.

We now present an additional, complementary approach, based on maximum likelihood analysis, which validates the functional form of *a*_*k*,*k*'_. The probability that out of *n*_*kk*' _pairs of duplicates with given connectivities *k *and *k*', *m*_*kk*' _pairs interact is , where *g*_*kk*' _gives the probability for a cross-interaction.  are the binomial coefficients. The probability *p *for observing for each pair *k *≤ *k*' *m*_*kk*' _interactions in *n*_*kk*' _pairs of duplicates is then given by . Symmetric and asymmetric attachment differ in how the probability of a cross-interaction *g*_*kk*' _depends on *k *and *k*'. In the symmetric case, *g*_*kk*' _= *g*_*k*_*g*_*k*'_. In the asymmetric case where one node is chosen uniformly, the other with a probability *f*_*k*_, we have *g*_*kk*' _= *f*_*k *_+ *f*_*k*'_. Using simulated annealing [31] we have calculated the (maximal) likelihoods *p *that the connectivity correlation pattern shown in fig. 3a resulted from either an asymmetric process, or a symmetric process, respectively, by maximizing *p *with respect to *f*_*k *_and *g*_*k*_. We find that the maximal likelihood for asymmetric attachment exceeds that for symmetric attachment by a factor *p*_asym_/*p*_sym _~ 4. The data thus favor an asymmetric attachment process, consistently with the biological interpretation given above. In addition, in the maximum likelihood analysis of the asymmetric model, *f*_*k *_shows an approximately linear increase with *k *(see figure 3c). Although this result is by no means conclusive, the data shows there is no reason to *a priori *consider only symmetric processes.

Thus far, we have only discussed the link attachment rate. For the detachment of links, we analogously assume that links are lost due to mutations at one of two linked nodes, and that the rate of this process does not depend on the properties of the other node that is unaffected by a mutation. The simplest mechanism reflecting these assumptions is one where a protein loses on average *d *links per unit time. A protein is chosen in an equiprobable manner from all nodes for removal of one of its links. The link to be removed is chosen at random from all its links. (An alternative detachment process consists in the loss of a certain *fraction *of links and leads to very similar results.) The resulting detachment rate is *d*_*k*,*k*' _~ (1/*k*) + (1/*k*'), where the inverse terms stem from nodes (rather than links) being chosen uniformly.

### Dynamical model of network evolution

The rates of the link dynamics discussed above, together with a slow growth of the network due to duplications, define a simple model for the evolution of protein interaction networks. Unlike previous models of the evolution of protein interaction networks [17-19] which emphasize the role of gene duplications, our model is based on the asymmetric link dynamics deduced from empirical data in the preceding section. By analytical solution or by numerical simulation one may investigate the networks generated by our model and compare their statistical properties to those of the empirical data on protein-interaction networks. This will be done in the present section. Before analyzing this model in the limit of large networks, we discuss the specific values of model parameters we used, and present the results of numerical simulations of a finite network.

We chose the initial network size such that after a suffcient waiting time, when a stationary state has been reached, the size of the simulated network matches that of the protein interaction data set we used (see methods). Duplication of nodes is modeled simply by adding new nodes with connectivity zero to the network at a rate of *g *= 10^-3 ^per node per million years, as motivated above. Using this simplistic growth mechanism is appropriate since, as shown above, the link dynamics will quickly alter the initial connectivity of a new node, as well as connectivity correlations with its neighbors. We begin with a total number of 4600 nodes, uniformly linked at random (giving a Poissonian connectivity distribution) such that the average connectivity of nodes with non-zero connectivity is *κ *= 2.5, the average connectivity found in the data set we used. After a waiting time of 25 million years there are 4696 nodes in total, of which 1872 nodes have non-zero connectivity. This is the size of the pooled protein interaction data set we used. The waiting time of 25 million years is of the same order of magnitude as the time scale on which connectivity correlations of duplicate nodes decay in figure 2a) of a few 10 million years.

New links are added at a rate of *a *= 0.59 new interactions per node per million years, using the asymmetric preferential linking rule we motivated above. Specifically, to form a new link we chose one node uniformly and a second node preferentially (i.e., with a probability proportional to its connectivity *k*) and link the two nodes. We removed links at a rate that keeps the average connectivity constant.

**Figure 4:**
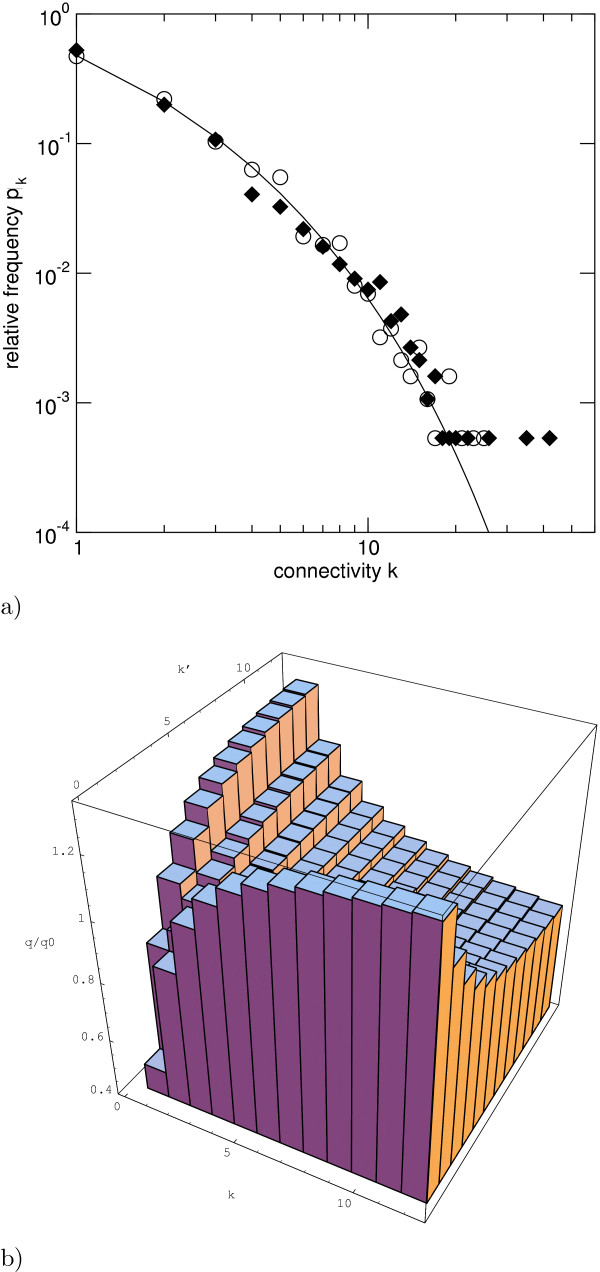
**(a) The asymmetric link dynamics produces a broad connectivity distribution. **The model prediction of the connectivity distribution of nodes with non-zero connectivity agrees well with yeast protein interaction data (filled diamonds). The solution of the rate equation (4) is shown as a solid line, the result of a computer simulation emulating the link dynamics encapsulated in (4) for a network of finite size is shown as circles (°). Nodes with the highest *k *(lower right) occur only once in the network. **(b) High-connectivity vertices are preferentially connected to low-connectivity vertices, as also observed empirically. **The figure shows the relative likelihood of the link distribution  and the 'null distribution'  of an uncorrelated random network, see text.

Specifically, at each time-step a link is deleted by choosing a node uniformly for link deletion if the average network connectivity exceeds *κ *= 2.5. The link to be deleted is chosen equiprobably from the links of this node. The connectivity distribution of a network whose evolution was simulated in this manner is shown in figure 4a) (open circles, °). This distribution is robust with respect to changes in the ratio of duplication to link dynamics *g/a *over at least an order of magnitude (results not shown).

We now turn to the consequences of this evolutionary dynamics for the statistical properties of the network. Since the link dynamics places and removes a link with a rate depending only on the connectivities of the nodes at either end, the evolutionary dynamics of the network can be represented in terms of the link connectivity distribution *q*_*k*,*k*'_. This distribution is defined as the fraction of network links that connect vertices of connectivities *k *and *k*',



where *c*_*ij *_= 1 if node *i *is linked to *j *and 0 otherwise. For convenience, a factor *κ *has been included in the normalization, i.e., ∑_*k*,*k*' _*q*_*k*,*k*' _= *κ*. The link connectivity distribution *q*_*k*,*k*' _captures correlations between the connectivities of neighboring vertices [23,32-34]. It is related to the single-vertex connectivity distribution by



for *k *> 0 and *p*_0 _= 1 - ∑_*k *> 0 _*p*_*k*_. The rates *a*_*k*,*k*' _and *d*_*k*,*k*' _are related to the total rates *a *and *d *of link detachment per unit time by the normalization



For a network of infinite size, link and growth dynamics result in a deterministic differential equation for the evolution of the link connectivity distribution *q*_*k*,*k*'_



The terms *J*_*k*,*k*' _arise from links that are not added or removed but that change their values (*k*,*k*'),



These are the links joining a mutated protein or its binding partner with third vertices, shown as open circles in fig. 1. The parameter *g *accounts for a uniform increase of the number of nodes caused by gene duplications.

In writing eq. (4), we have assumed that next-nearest neighbor connectivity correlations vanish. This assumption is self-consistent since the stationary solution has indeed only nearest-neighbor correlations. Truncating all correlations and writing down an evolution equation for the connectivity distribution *p*_*k *_turns out to be inconsistent since asymmetric link dynamics generates non-trivial connectivity correlations. This distinguishes the present model from simpler models of network growth, which can be self-consistently formulated at the level of the distribution *p*_*k*_.

We solved equation eq. (4), which describes the evolution of the connectivity correlations numerically for its steady state. For initial conditions we use a Poissonian connectivity distribution where the average connectivity of connected nodes is 2.5, and connectivity correlations which factorize *q*_*k*,*k*' _~ *kk*'*p*_*k*_*p*_*k*_'. We followed the time evolution of *q*_*k*,*k*' _defined by eq. (4) until a steady state was reached using the parameters *a *and *g *given above and choosing *d *such that the average connectivity of connected nodes remains at a constant *κ *= 2.5. This procedure leads to a stationary link connectivity distribution  and a resulting connectivity distribution  independent of initial conditions. Because the evolution equation is a rate-equation that applies to a network of infinite size, the parameters determining the stationary state are the ratio between growth and attachment rate, the functional form of the attachment and detachment rates, and the average connectivity. The stationary state turns out to be asymptotically independent of the duplication rate for small duplication rates. In fact, if we solve eq. (4) numerically for any ratio *g*/*a *< 10^-1^, the results are statistically indistinguishable from that for *g *= 0, implying great robustness against errors in the rate estimates discussed above.

The statistical properties of our model in its stationary state may now be compared with the corresponding quantities in the protein-interaction network. The connectivity distribution  agrees well with the empirical data as shown in fig. 4(a) along with the results of numerical simulations. The distribution is broad but not scale free. (From the empirical data with connectivities distributed over little more than a single decade the scale-free property of protein networks – meaning that connectivities are distributed according to a power law – can not be confidently ascertained. Furthermore the empirical data shown in fig. 4 distinctly deviates from a power-law.) This also holds for uniform detachment, where *d*_*kk*' _= constant, and it is a crucial difference to models with symmetric attachment, where preferential attachment leads to scale-free networks, both at constant network size [30], and in growing networks [3,35].

For the connectivity correlations, we find that vertices of high k are more frequently linked to vertices of low *k*' than in an uncorrelated random network with the same connectivity distribution . Fig. 4(b) shows the relative likelihood  is the link connectivity distribution of the network with no connectivity correlations. Correlations with this property have recently been reported for the protein interaction network in yeast [23], but a quantitative comparison with the prediction of our model will have to await a greater amount of reliable protein interaction data. We note that connectivity correlations are a specific property of networks shaped by asymmetric dynamics, and are absent in the case of symmetric dynamics, as discussed in the appendix. In other words, the empirically observed non-trivial connectivity correlations require asymmetric link dynamics. This is an *a posteriori *reason for considering asymmetric link dynamics.

A further consequence of asymmetric attachment is that our model does not obey detailed balance (as is the case of symmetric link dynamics, where attachment and detachment rates do factorize, see [30]). Asymmetric attachment or detachment rules violate the condition, necessary for detailed balance, that the product of transition probabilities along a circular trajectory in the space of networks is independent of the direction of this tour. This may be demonstrated easily by considering, e.g. four nodes labeled 1 – 4 to be connected linearly and disconnected again. Starting and ending with a single link between nodes (1, 2), say, the product of the rates of adding a link between (2, 3), then (3, 4) before removing the links between (2, 3) and then (3, 4) is , that for the same tour in reverse is , which are generally equal only if the rates facorize in their arguments.

## Conclusions

We have presented a stochastic evolution model for protein networks, which is based on fast link dynamics due to mutations of the coding sequence of existing proteins and a slow growth dynamics through gene duplications. The crucial ingredient of the link dynamics is an asymmetric preferential attachment rule, which is supported by empirical data. The asymmetry has a simple biological interpretation, namely that mutations in one gene may lead to a new interaction of its product with that of another, unchanged, gene. Such a mechanism, where the two nodes involved in the generation of a new link play different roles, is probably the norm, rather than the exception, in biological networks. This holds particularly for regulatory networks, where a new interaction between two genes is formed by changes in the regulatory region of only *one *of them.

Asymmetric link dynamics leads to a network model, where the aggregate variables necessary to describe network structure are the connectivity correlations *q*_*k*,*k*'_, which give the fraction of links with connectivities *k *and *k*'. In our case, the model successfully reproduces the connectivity distribution found in empirically available protein interaction data. The asymmetry of the link dynamics also leads to connectivity correlations between interacting proteins, which have been observed empirically [23]. A model with symmetric link dynamics, on the other hand, produces no such correlations. Higher order correlations of this kind [33] are of particular interest for future work as they may be a quantitative signature of natural selection on the level of the network as a whole.

## Methods

### Data processing

The protein interaction data in this paper was pooled from three sources. The first of these sources is a large-scale high-throughput experiment using the yeast two-hybrid assay [13] (data available from [41]). It comprises 899 pairwise interactions among 985 proteins. The second source is also a high-throughput two-hybrid experiment, from which we used a "core" set of 747 interactions between 780 proteins, interactions that had been confirmed through replicated experiments [9,42]. The third source is the public MIPS database [36,43] of May 2001. From this database, we included only pairwise interactions that were not produced by the two-hybrid assay, but instead by other techniques such as cross-linking or co-purification of two proteins. This resulted in 899 interactions between 680 proteins After pooling the three data-sets and eliminating redundant interactions, we were left with a network of 2463 interactions and 1893 proteins.

While enormously valuable in their own right, analyses of protein complexes do not identify pairwise protein interactions, and were thus not suitable for our analysis [7,8]. We also excluded interaction data derived from experiments identifying domain-specific rather than whole-protein interactions [10-12]. For all three data sets taken separately, the connectivity distributions are statistically indistinguishable [22]. Moreover, the observations on link addition we use here [22], as well as the patterns in Fig. 2 hold qualitatively for each data set individually.

Data on yeast gene duplicates, generated as described in [27], was kindly provided by John Conery (University of Oregon, Department of Computer Science). Briefly, gapped BLAST [37] was used for pairwise amino acid sequence comparisons of all yeast open reading frames as obtained from GenBank. All protein pairs with a BLAST alignment score greater than 10^-2^were retained for further analysis. Then, the following conservative approach was taken to retain only unambiguously aligned sequences: Using the protein alignment generated by BLAST as a guide, a sequence pair was scanned to the right of each alignment gap. The part of the sequence from the end of the gap to the first "anchor" pair of matched amino acids was discarded. The remaining sequence (apart from the anchor pair of amino acids) was retained if a second pair of matching amino acids was found within less than six amino acids from the first. This procedure was then repeated to the left of each alignment gap (see [27] for more detailed description and justification). The retained portion of each amino acid sequence alignment was then used jointly with DNA sequence information to generate nucleotide sequence alignments of genes. For each gene pair in this data set, the fraction *K*_*s *_of synonymous (silent) substitutions per silent site, as well as the fraction *K*_*a *_of replacement substitutions per replacement site were estimated using the method of Li [28].

### Asymmetric link dynamics and connectivity correlations

The existence of non-trivial correlations may be attributed directly to the asymmetry of the link dynamics. Symmetric link dynamics, which is a standard mechanism in models of networks at constant size [30], leads to networks with uncorrelated connectivities: Generalizing the approach of [30] to include connectivity-dependent detachment, one obtains for symmetric link dynamics with rates *a*_*k *_and *d*_*k *_an equilibrium distribution giving the probability of finding the network in the state given by adjacency matrix *c*_*ij *_of . This results in a connectivity distribution  and trivial connectivity correlations , which factorize in the connectivities. This results ina constant . A model with symmetric link dynamics can thus produce any empirically observed connectivity distribution, but no networks with statistically significant connectivity correlations.

## Authors' contributions

ML and AW contributed equally to this work. All authors read and approved the final manuscript.
